# Gunshot Wound of Abdomen with Twelve Perforations—Recovery

**Published:** 1898-11

**Authors:** W. Fisher Cole

**Affiliations:** Pendleton, Ore.


### GUNSHOT WOUND OF ABDOMEN, WITH TWELVE PERFORATIONS—RECOVERY.

By W. Fisher Cole, M. D., Pendleton, Ore.

At 12 o'clock on the morning of July 2d I was summoned by Dr. F. W. Vincent to see G. E., who a few minutes previously had been shot through the abdomen by a .38 calibre pistol. The man was ordered to my private hospital. The legal authorities and a clergyman summoned, at the patient's request, as the indications clearly pointed to a serious condition within the abdominal cavity, there being quite a considerable hemorrhage from the wound of entrance and marked distension of the abdomen.

The patient was hastily prepared for operation by the usual aseptic method. The wound of entrance was located two inches below and three inches to right of umbilicus.

The point at which the bullet was removed was one-half inch above and six inches to left of umbilicus.

The abdomen was opened in the median line with escape of considerable blood and fecal matter. On bringing up a loop of intestine, a perforated portion was found, cleansed, sutured and returned. This method was followed until twelve peforations had been closed. In two places the bullet had passed along the length of the intestine, tearing ragged holes an inch in length, severing the mesentric artery, from which blood spurted at every impulse of the heart.

The bleeding points in the mesentery were tied with silk and the perforations closed by silk suture.

The Murphy button was not used, owing to the fact that it would have necessitated an extensive resection, and also my previous successful experience with a gunshot wound of the abdomen with four perforations, led me to hope for a more favorable result in the use of the Lembert suture.

Twice during the operation work had to be suspended on account of the embarrassed respiration, it being necessary to carry the anæsthetic to an extreme degree to quiet the spasmodic condition present, and to control the escape of intestines from the wound.

The openings closed, bleeding surfaces ligated, the peritoneal cavity was cleansed first by means of steriled gauze pads, then flushed with hot saline solution, and again carefully sponged, until the pads returned practically dry and unstained. The abdominal wound was then closed by means of silkworm-gut sutures.

The patient was removed from the table with a pulse of 160 and evidence of considerable shock. Artificial heat was applied and in two hours he had rallied sufficiently to ask concerning the operation. No food or drink was allowed for twelve hours, at the expiration of this time the patient was allowed small amounts of Trophonine and ice water, with a few drops of essence of Peppermint. The food for the first fifteen days consisted of Trophonine and malted milk.

Action from the bowels occurred on the afternoon of the fourth day after the operation. The highest temperature was 100½ on the morning of the second day. Wound healed by first intention.

Patient left hospital four weeks from date of operation without any apparent inconvenience from his experience.

I was ably assisted in the operation by Dr. F. W. Vincent, of this city.—*Medical Sentinel for October.*

